# (1*R*,3*S*)-Methyl 2-benzyl-6,7-dimeth­oxy-1-phenyl-1,2,3,4-tetra­hydro­isoquinoline-3-carboxyl­ate

**DOI:** 10.1107/S1600536809050521

**Published:** 2009-11-28

**Authors:** Tricia Naicker, Michael McKay, Thavendran Govender, Hendrik G. Kruger, Glenn E. M. Maguire

**Affiliations:** a School of Chemistry, University of KwaZulu-Natal, Durban 4000, South Africa; b School of Pharmacy and Pharmacology, University of KwaZulu-Natal, Durban 4000, South Africa

## Abstract

In the title compound, C_26_H_27_NO_4_, a precursor to novel chiral catalysts, the N-containing six-membered ring assumes a half-boat conformation. Various C—H⋯π interactions and intermolecular short contacts (C⋯H = 2.81–2.90 Å) link the mol­ecules together in the crystal structure.

## Related literature

For the synthesis, see: Chakka *et al.* (2009 ▶). For crystallograhic details of analogous mol­ecules, see Alberch *et al.* (2004 ▶); Aubry *et al.* (2006 ▶).
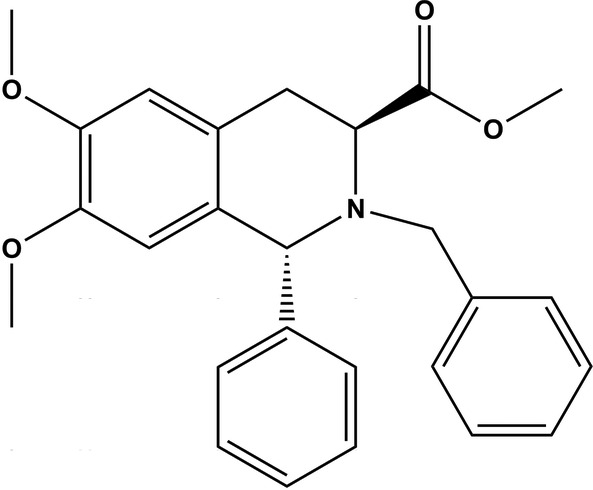


## Experimental

### 

#### Crystal data

C_26_H_27_NO_4_
*M* *_r_* = 417.49
Triclinic, 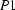

*a* = 6.0199 (1) Å
*b* = 9.2592 (2) Å
*c* = 11.0429 (2) Å
α = 73.365 (1)°
β = 74.694 (1)°
γ = 75.737 (1)°
*V* = 559.05 (2) Å^3^
*Z* = 1
Cu *K*α radiation
μ = 0.67 mm^−1^
*T* = 173 K
0.22 × 0.12 × 0.08 mm

#### Data collection

Bruker Kappa Duo APEXII diffractometer
Absorption correction: multi-scan (*SADABS*; Sheldrick, 1997 ▶) *T*_min_ = 0.692, *T*_max_ = 0.753
7546 measured reflections
3561 independent reflections
3536 reflections with *I* > 2σ(*I*)
*R*_int_ = 0.012

#### Refinement

*R*[*F* ^2^ > 2σ(*F* ^2^)] = 0.028
*wR*(*F* ^2^) = 0.077
*S* = 1.07
3561 reflections
281 parameters
3 restraints
H-atom parameters constrained
Δρ_max_ = 0.16 e Å^−3^
Δρ_min_ = −0.16 e Å^−3^
Absolute structure: Flack (1983 ▶), 1483 Friedel pairs
Flack parameter: −0.01 (14)

### 

Data collection: *APEX2* (Bruker, 2006 ▶); cell refinement: *SAINT* (Bruker, 2006 ▶); data reduction: *SAINT*; program(s) used to solve structure: *SHELXS97* (Sheldrick, 2008 ▶); program(s) used to refine structure: *SHELXL97* (Sheldrick, 2008 ▶) and *X-SEED* (Barbour, 2001 ▶); molecular graphics: *ORTEP-3* (Farrugia, 1997 ▶); software used to prepare material for publication: *ORTEP-3*.

## Supplementary Material

Crystal structure: contains datablocks I, global. DOI: 10.1107/S1600536809050521/hg2607sup1.cif

Structure factors: contains datablocks I. DOI: 10.1107/S1600536809050521/hg2607Isup2.hkl

Additional supplementary materials:  crystallographic information; 3D view; checkCIF report

## Figures and Tables

**Table 1:** C—H⋯π interaction (Å, °)

*D*—H⋯*A*	*D*—H	H⋯*A*	*D*⋯*A*	*D*—H⋯*A*
C19—H19*A*⋯*Cg* ^i^	0.98	2.82	3.639 (2)	148

Symmetry code: (i) 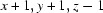. *Cg* is the centroid of the C21–C26 ring.

**Fig. 1.**
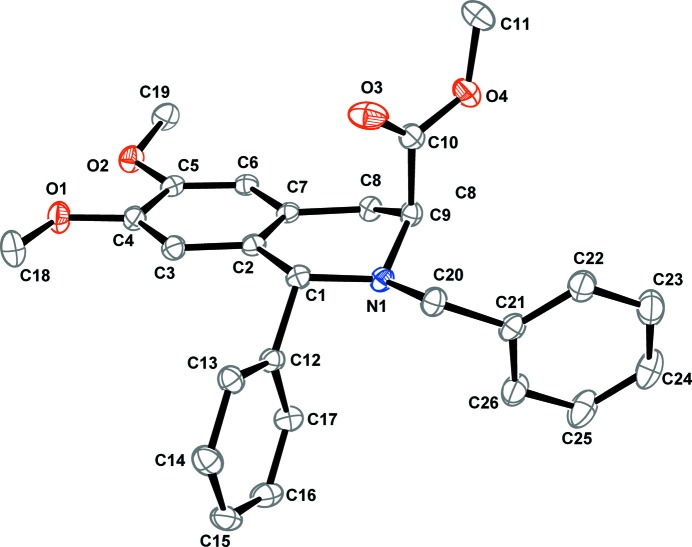
Molecular structure of the title compound 2 showing numbering scheme. Displacement elipsoids are drawn at the 30% probability level. Hydrogen atoms are omitted for clarity.

**Fig. 2.**
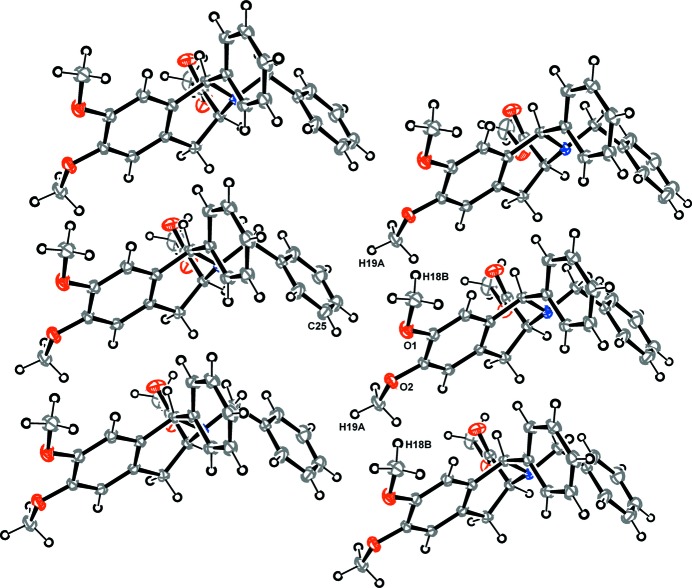
Intermolecular short contact interactions present in the crystal structure of the title compound 2. The atoms involved in the interactions (as discussed) are labelled. different interactions displayed by methoxy groups. Displacement ellipsoids are drawn at the 30% probability level, and the hydrogen atoms appear as speheres of arbitrary radii.

**Fig. 3.**
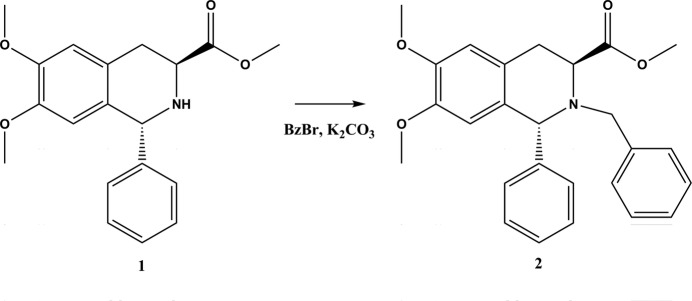
The formation of the title compound.

## supplementary crystallographic information

### Comment

The title compound (2, Scheme 1) is a precursor in the synthesis of novel chiral ligands containing a tetrahydroisoquinoline backbone. We have recently reported the use of these ligands as successful catalysts for transfer hydrogenations reactions (Chakka *et al.*, 2009).

Compound 2 was derived from commercially available *L*-DOPA and benzaldehyde. Diastereomers formed during the first step of the synthesis were separated to yield subsequent derivatives and the title compound and with the stereochemistry as illustrated in Figure 1. The absolute stereochemistry was confirmed to be *R,S* at C1 and C9 positions respectively.

From the crystal structure it is evident that the N-containing six membered ring assumes a half boat conformation (Figure 1). This differs from an analogous structure which assumes a half chair conformation (Aubry *et al.*, 2006 and Alberch *et al.*, 2004). A possible reason for this change in conformation could be either the introduction of a substitutent on the nitrogen or the *trans* position of the phenyl ring at the C1 position.

The molecule exhibits various intermolecular short contacts *i.e*. between the methyl ester hydrogen (H11C) and phenyl ring (C14) of a neighbouring molecule; H15 to C6 and C7 and H24 to C14 and C15.

The methoxy groups display different interactions. The first methoxy group at C4 position displays one interaction between H18B and O2, which is the ether oxygen of the other methoxy group. The second methoxy group at C5 position displays three interactions; the first being the above mentioned interaction with H18B and O2, the second being a short contact between O2 and H25, and the third being another CH-*π* the interaction between H19A and C25. The atoms involved in these short contacts are shown in Figure 2.

### Experimental

To a solution of 1 (Scheme 1) (500 mg, 1.52 mmol) in acetonitrile (20 ml), solid K_2_CO_3 _(635 mg, 4.58 mmol) was added followed by benzyl bromide (286 mg, 1.67 mmol) at ambient temperature. There after the reaction mixture was refluxed for 3 h. Completion of the reaction was monitored with TLC using hexane/ethyl acetate (60:40, *R*_f_ 1/2). The solvent was evaporated and 30 ml of ethylacetate was added, washed with 2 × 10 ml of water, the organic layer was separated, and dried over anhydrous MgSO_4_. The solvent was evaporated under reduced pressure to afford crude product, which was purified by column chromatography using hexane/ethyl acetate (60:40) as the eluent to yield pure benzyl ester 2 (0.44 g, 90%) as a white solid.

^1^H NMR (400 MHz/CDCl_3_): δ = 7.44 (d, *J* = 1.16 Hz, 2H), 7.32–7.10 (m, 14H), 7.0–6.88 (m, 6H), 6.69 (s, 1H), 6.38 (s, 1H), 4.74 (s,1*H*), 4.21 (d, *J* = 13.60 Hz, 1H), 4.14 (q, *J* = 3.70, 12.74 Hz, 1H), 3.89 (s, 3H), 3.72 (s, 3H), 3.57 (s, 1H), 3.30–3.18 (m, 2H), 2.60 (dd, *J* = 3.60, 16.48 Hz, 1H).

Colourless crystals suitable for X-ray diffraction were obtained by slow evaporation of 2 in dichloromethane at room temperature.

### Refinement

Hydrogen atoms, first located in the difference map, were positioned geometrically and allowed to ride on their respective parent atoms, with C—H bond lengths of 1.00 (CH), 0.99 (CH_2_), 0.98 (CH_3_) or 0.93 (aromatic CH). They were then refined with a riding model with *U*_iso_(H) = 1.5*U*_eq_(CH_3_) and *U*_iso_(H) = 1.2*U*_eq_(*X*) for *X* = CH or CH_2_. The largest residual electron density peak of 0.16 e/Å^3^ is 0.86 Å from O4.

### Crystal data

**Table:**

C_26_H_27_NO_4_	*Z* = 1
*M**_r_* = 417.49	*F*(000) = 222
Triclinic, *P*1	*D*_x_ = 1.240 Mg m^−^^3^
Hall symbol: P 1	Melting point: 420 K
*a* = 6.0199 (1) Å	Cu *K*α radiation, λ = 1.54184 Å
*b* = 9.2592 (2) Å	Cell parameters from 7546 reflections
*c* = 11.0429 (2) Å	θ = 4.3–68.9°
α = 73.365 (1)°	µ = 0.67 mm^−^^1^
β = 74.694 (1)°	*T* = 173 K
γ = 75.737 (1)°	Needle, yellow
*V* = 559.04 (2) Å^3^	0.22 × 0.12 × 0.08 mm

### Data collection

**Table:**

Bruker Kappa Duo APEXII diffractometer	3561 independent reflections
Radiation source: fine-focus sealed tube	3536 reflections with *I* > 2σ(*I*)
graphite	*R*_int_ = 0.012
0.5° φ scans and ω scans	θ_max_ = 68.9°, θ_min_ = 4.3°
Absorption correction: multi-scan (*SADABS*; Sheldrick, 1997)	*h* = −6→7
*T*_min_ = 0.692, *T*_max_ = 0.753	*k* = −10→10
7546 measured reflections	*l* = −13→13

### Refinement

**Table:**

Refinement on *F*^2^	Hydrogen site location: inferred from neighbouring sites
Least-squares matrix: full	H-atom parameters constrained
*R*[*F*^2^ > 2σ(*F*^2^)] = 0.028	*w* = 1/[σ^2^(*F*_o_^2^) + (0.0475*P*)^2^ + 0.0785*P*] where *P* = (*F*_o_^2^ + 2*F*_c_^2^)/3
*wR*(*F*^2^) = 0.077	(Δ/σ)_max_ < 0.001
*S* = 1.07	Δρ_max_ = 0.16 e Å^−^^3^
3561 reflections	Δρ_min_ = −0.16 e Å^−^^3^
281 parameters	Extinction correction: *SHELXS97* (Sheldrick, 2008), Fc^*^=kFc[1+0.001xFc^2^λ^3^/sin(2θ)]^-1/4^
3 restraints	Extinction coefficient: 0.0426 (18)
Primary atom site location: structure-invariant direct methods	Absolute structure: Flack (1983), 1483 Friedel pairs
Secondary atom site location: difference Fourier map	Flack parameter: −0.01 (14)

### Special details

**Table:**

Experimental. Half sphere of data collected using *SAINT* strategy (Bruker, 2006). Crystal to detector distance = 50 mm; combination of φ and ω scans of 0.5°, 80 s per °, 2 iterations.
Geometry. All e.s.d.'s (except the e.s.d. in the dihedral angle between two l.s. planes) are estimated using the full covariance matrix. The cell e.s.d.'s are taken into account individually in the estimation of e.s.d.'s in distances, angles and torsion angles; correlations between e.s.d.'s in cell parameters are only used when they are defined by crystal symmetry. An approximate (isotropic) treatment of cell e.s.d.'s is used for estimating e.s.d.'s involving l.s. planes.
Refinement. Refinement of *F*^2^ against ALL reflections. The weighted *R*-factor *wR* and goodness of fit *S* are based on *F*^2^, conventional *R*-factors *R* are based on *F*, with *F* set to zero for negative *F*^2^. The threshold expression of *F*^2^ > σ(*F*^2^) is used only for calculating *R*-factors(gt) *etc*. and is not relevant to the choice of reflections for refinement. *R*-factors based on *F*^2^ are statistically about twice as large as those based on *F*, and *R*- factors based on ALL data will be even larger.

### Fractional atomic coordinates and isotropic or equivalent isotropic displacement parameters (Å^2^)

**Table:**

	*x*	*y*	*z*	*U*_iso_*/*U*_eq_	
O1	0.2537 (2)	0.14310 (13)	0.46249 (10)	0.0396 (3)	
O2	0.51646 (19)	0.27489 (12)	0.53134 (10)	0.0364 (3)	
O3	−0.3602 (2)	0.20138 (13)	1.02179 (14)	0.0468 (3)	
O4	−0.1914 (3)	0.23948 (15)	1.16480 (12)	0.0514 (4)	
N1	−0.0675 (2)	−0.10502 (13)	1.05031 (11)	0.0262 (3)	
C1	−0.1027 (2)	−0.07812 (15)	0.91791 (14)	0.0247 (3)	
H1	−0.2641	−0.0179	0.9133	0.030*	
C2	0.0745 (2)	0.01232 (15)	0.81875 (14)	0.0256 (3)	
C3	0.0848 (3)	0.03078 (16)	0.68640 (14)	0.0283 (3)	
H3	−0.0128	−0.0169	0.6612	0.034*	
C4	0.2340 (3)	0.11668 (16)	0.59283 (14)	0.0298 (3)	
C5	0.3790 (2)	0.18793 (15)	0.63010 (14)	0.0280 (3)	
C6	0.3717 (2)	0.16708 (16)	0.75934 (14)	0.0270 (3)	
H6	0.4725	0.2122	0.7846	0.032*	
C7	0.2187 (2)	0.08063 (15)	0.85461 (13)	0.0252 (3)	
C8	0.2148 (2)	0.06332 (17)	0.99497 (14)	0.0292 (3)	
H8A	0.2370	0.1606	1.0067	0.035*	
H8B	0.3468	−0.0180	1.0206	0.035*	
C9	−0.0138 (3)	0.02263 (16)	1.08230 (14)	0.0269 (3)	
H9	0.0152	−0.0155	1.1721	0.032*	
C10	−0.2107 (3)	0.16235 (17)	1.08378 (14)	0.0308 (3)	
C11	−0.3714 (5)	0.3725 (2)	1.1814 (2)	0.0633 (6)	
H11A	−0.3393	0.4199	1.2422	0.095*	
H11B	−0.5244	0.3412	1.2153	0.095*	
H11C	−0.3725	0.4466	1.0979	0.095*	
C12	−0.0842 (3)	−0.23317 (16)	0.89052 (13)	0.0263 (3)	
C13	−0.2621 (3)	−0.26446 (17)	0.84954 (14)	0.0293 (3)	
H13	−0.3966	−0.1876	0.8363	0.035*	
C14	−0.2445 (3)	−0.40782 (18)	0.82777 (16)	0.0346 (3)	
H14	−0.3673	−0.4285	0.7998	0.042*	
C15	−0.0496 (3)	−0.52049 (17)	0.84649 (16)	0.0368 (4)	
H15	−0.0378	−0.6184	0.8315	0.044*	
C16	0.1286 (3)	−0.48936 (17)	0.88734 (16)	0.0382 (4)	
H16	0.2632	−0.5662	0.9002	0.046*	
C17	0.1116 (3)	−0.34743 (17)	0.90937 (16)	0.0330 (3)	
H17	0.2344	−0.3274	0.9377	0.040*	
C18	0.1052 (3)	0.0782 (2)	0.41938 (17)	0.0462 (4)	
H18A	0.1349	0.1062	0.3247	0.069*	
H18B	−0.0591	0.1176	0.4541	0.069*	
H18C	0.1379	−0.0337	0.4497	0.069*	
C19	0.6262 (3)	0.3736 (2)	0.56531 (18)	0.0429 (4)	
H19A	0.7198	0.4294	0.4872	0.064*	
H19B	0.7283	0.3123	0.6249	0.064*	
H19C	0.5059	0.4469	0.6072	0.064*	
C20	−0.2513 (3)	−0.17390 (17)	1.15037 (14)	0.0304 (3)	
H20A	−0.2927	−0.2542	1.1219	0.037*	
H20B	−0.3934	−0.0944	1.1635	0.037*	
C21	−0.1694 (3)	−0.24341 (17)	1.27560 (14)	0.0311 (3)	
C22	−0.2758 (3)	−0.1877 (2)	1.38479 (16)	0.0404 (4)	
H22	−0.4093	−0.1075	1.3826	0.049*	
C23	−0.1904 (4)	−0.2472 (2)	1.49690 (17)	0.0506 (5)	
H23	−0.2661	−0.2086	1.5714	0.061*	
C24	0.0038 (4)	−0.3620 (2)	1.50087 (18)	0.0536 (5)	
H24	0.0642	−0.4016	1.5775	0.064*	
C25	0.1101 (4)	−0.4193 (3)	1.39405 (19)	0.0553 (5)	
H25	0.2436	−0.4994	1.3971	0.066*	
C26	0.0240 (3)	−0.3612 (2)	1.28165 (17)	0.0442 (4)	
H26	0.0980	−0.4022	1.2082	0.053*	

### Atomic displacement parameters (Å^2^)

**Table:**

	*U*^11^	*U*^22^	*U*^33^	*U*^12^	*U*^13^	*U*^23^
O1	0.0486 (7)	0.0478 (7)	0.0253 (5)	−0.0190 (5)	−0.0072 (5)	−0.0048 (5)
O2	0.0368 (6)	0.0380 (6)	0.0331 (6)	−0.0168 (5)	−0.0028 (5)	−0.0019 (4)
O3	0.0365 (6)	0.0370 (6)	0.0757 (9)	0.0013 (5)	−0.0246 (6)	−0.0222 (6)
O4	0.0722 (10)	0.0442 (7)	0.0412 (6)	0.0050 (6)	−0.0192 (6)	−0.0217 (5)
N1	0.0261 (6)	0.0264 (6)	0.0261 (6)	−0.0075 (5)	−0.0070 (5)	−0.0023 (5)
C1	0.0222 (7)	0.0244 (7)	0.0275 (7)	−0.0042 (5)	−0.0065 (6)	−0.0050 (5)
C2	0.0230 (7)	0.0222 (6)	0.0291 (7)	−0.0020 (5)	−0.0053 (6)	−0.0044 (5)
C3	0.0290 (7)	0.0276 (7)	0.0295 (7)	−0.0058 (6)	−0.0086 (6)	−0.0058 (6)
C4	0.0312 (8)	0.0294 (8)	0.0275 (7)	−0.0033 (6)	−0.0076 (6)	−0.0052 (6)
C5	0.0250 (7)	0.0236 (7)	0.0311 (7)	−0.0033 (5)	−0.0040 (6)	−0.0027 (5)
C6	0.0230 (7)	0.0245 (7)	0.0337 (8)	−0.0024 (5)	−0.0085 (6)	−0.0065 (6)
C7	0.0228 (7)	0.0222 (7)	0.0297 (7)	−0.0006 (5)	−0.0088 (6)	−0.0049 (6)
C8	0.0263 (8)	0.0325 (7)	0.0302 (7)	−0.0075 (6)	−0.0081 (6)	−0.0058 (6)
C9	0.0275 (7)	0.0291 (7)	0.0250 (6)	−0.0072 (5)	−0.0069 (6)	−0.0050 (5)
C10	0.0316 (8)	0.0305 (7)	0.0298 (7)	−0.0109 (6)	−0.0013 (6)	−0.0065 (6)
C11	0.0849 (16)	0.0475 (11)	0.0494 (11)	0.0069 (10)	−0.0024 (11)	−0.0257 (9)
C12	0.0268 (7)	0.0247 (7)	0.0253 (7)	−0.0068 (5)	−0.0022 (6)	−0.0040 (5)
C13	0.0283 (8)	0.0314 (7)	0.0285 (7)	−0.0065 (6)	−0.0038 (6)	−0.0085 (6)
C14	0.0344 (8)	0.0366 (8)	0.0369 (8)	−0.0141 (6)	−0.0029 (7)	−0.0126 (6)
C15	0.0429 (9)	0.0267 (7)	0.0385 (8)	−0.0109 (7)	0.0038 (7)	−0.0115 (6)
C16	0.0353 (8)	0.0269 (8)	0.0444 (9)	−0.0015 (6)	−0.0021 (7)	−0.0057 (6)
C17	0.0285 (7)	0.0303 (8)	0.0387 (8)	−0.0053 (6)	−0.0058 (6)	−0.0069 (6)
C18	0.0482 (10)	0.0640 (12)	0.0332 (8)	−0.0173 (8)	−0.0102 (8)	−0.0148 (8)
C19	0.0440 (9)	0.0401 (9)	0.0446 (9)	−0.0217 (7)	−0.0066 (8)	−0.0005 (7)
C20	0.0289 (7)	0.0335 (7)	0.0287 (7)	−0.0100 (6)	−0.0052 (6)	−0.0042 (6)
C21	0.0307 (8)	0.0308 (8)	0.0297 (7)	−0.0122 (6)	−0.0048 (6)	0.0001 (6)
C22	0.0433 (9)	0.0407 (9)	0.0340 (8)	−0.0071 (7)	−0.0067 (7)	−0.0055 (7)
C23	0.0641 (13)	0.0568 (11)	0.0323 (8)	−0.0186 (9)	−0.0081 (8)	−0.0083 (8)
C24	0.0515 (11)	0.0696 (13)	0.0350 (9)	−0.0169 (10)	−0.0173 (8)	0.0073 (8)
C25	0.0405 (10)	0.0633 (12)	0.0423 (10)	0.0024 (8)	−0.0087 (8)	0.0080 (9)
C26	0.0414 (10)	0.0460 (10)	0.0322 (8)	0.0004 (7)	−0.0032 (7)	−0.0005 (7)

### Geometric parameters (Å, °)

**Table:**

O1—C4	1.3668 (18)	C12—C17	1.393 (2)
O1—C18	1.428 (2)	C13—C14	1.390 (2)
O2—C5	1.3681 (17)	C13—H13	0.9500
O2—C19	1.428 (2)	C14—C15	1.382 (2)
O3—C10	1.197 (2)	C14—H14	0.9500
O4—C10	1.3363 (19)	C15—C16	1.385 (3)
O4—C11	1.446 (2)	C15—H15	0.9500
N1—C9	1.4538 (18)	C16—C17	1.379 (2)
N1—C20	1.4667 (18)	C16—H16	0.9500
N1—C1	1.4743 (18)	C17—H17	0.9500
C1—C12	1.5222 (19)	C18—H18A	0.9800
C1—C2	1.5285 (19)	C18—H18B	0.9800
C1—H1	1.0000	C18—H18C	0.9800
C2—C7	1.381 (2)	C19—H19A	0.9800
C2—C3	1.408 (2)	C19—H19B	0.9800
C3—C4	1.378 (2)	C19—H19C	0.9800
C3—H3	0.9500	C20—C21	1.505 (2)
C4—C5	1.412 (2)	C20—H20A	0.9900
C5—C6	1.375 (2)	C20—H20B	0.9900
C6—C7	1.402 (2)	C21—C22	1.384 (2)
C6—H6	0.9500	C21—C26	1.387 (2)
C7—C8	1.506 (2)	C22—C23	1.381 (3)
C8—C9	1.521 (2)	C22—H22	0.9500
C8—H8A	0.9900	C23—C24	1.374 (3)
C8—H8B	0.9900	C23—H23	0.9500
C9—C10	1.525 (2)	C24—C25	1.369 (3)
C9—H9	1.0000	C24—H24	0.9500
C11—H11A	0.9800	C25—C26	1.385 (3)
C11—H11B	0.9800	C25—H25	0.9500
C11—H11C	0.9800	C26—H26	0.9500
C12—C13	1.386 (2)		
			
C4—O1—C18	117.43 (12)	C17—C12—C1	119.97 (13)
C5—O2—C19	116.91 (12)	C12—C13—C14	120.24 (14)
C10—O4—C11	116.59 (16)	C12—C13—H13	119.9
C9—N1—C20	111.85 (11)	C14—C13—H13	119.9
C9—N1—C1	116.21 (11)	C15—C14—C13	120.45 (15)
C20—N1—C1	113.54 (11)	C15—C14—H14	119.8
N1—C1—C12	108.07 (11)	C13—C14—H14	119.8
N1—C1—C2	111.52 (11)	C14—C15—C16	119.39 (14)
C12—C1—C2	111.33 (11)	C14—C15—H15	120.3
N1—C1—H1	108.6	C16—C15—H15	120.3
C12—C1—H1	108.6	C17—C16—C15	120.36 (14)
C2—C1—H1	108.6	C17—C16—H16	119.8
C7—C2—C3	119.04 (13)	C15—C16—H16	119.8
C7—C2—C1	122.20 (12)	C16—C17—C12	120.60 (15)
C3—C2—C1	118.73 (13)	C16—C17—H17	119.7
C4—C3—C2	121.22 (14)	C12—C17—H17	119.7
C4—C3—H3	119.4	O1—C18—H18A	109.5
C2—C3—H3	119.4	O1—C18—H18B	109.5
O1—C4—C3	125.46 (14)	H18A—C18—H18B	109.5
O1—C4—C5	115.04 (12)	O1—C18—H18C	109.5
C3—C4—C5	119.50 (13)	H18A—C18—H18C	109.5
O2—C5—C6	125.15 (14)	H18B—C18—H18C	109.5
O2—C5—C4	115.76 (13)	O2—C19—H19A	109.5
C6—C5—C4	119.09 (13)	O2—C19—H19B	109.5
C5—C6—C7	121.42 (14)	H19A—C19—H19B	109.5
C5—C6—H6	119.3	O2—C19—H19C	109.5
C7—C6—H6	119.3	H19A—C19—H19C	109.5
C2—C7—C6	119.71 (13)	H19B—C19—H19C	109.5
C2—C7—C8	120.93 (12)	N1—C20—C21	110.54 (12)
C6—C7—C8	119.36 (13)	N1—C20—H20A	109.5
C7—C8—C9	112.16 (12)	C21—C20—H20A	109.5
C7—C8—H8A	109.2	N1—C20—H20B	109.5
C9—C8—H8A	109.2	C21—C20—H20B	109.5
C7—C8—H8B	109.2	H20A—C20—H20B	108.1
C9—C8—H8B	109.2	C22—C21—C26	118.49 (15)
H8A—C8—H8B	107.9	C22—C21—C20	121.50 (15)
N1—C9—C8	109.50 (12)	C26—C21—C20	119.94 (15)
N1—C9—C10	115.01 (12)	C23—C22—C21	120.77 (17)
C8—C9—C10	112.26 (12)	C23—C22—H22	119.6
N1—C9—H9	106.5	C21—C22—H22	119.6
C8—C9—H9	106.5	C24—C23—C22	120.14 (18)
C10—C9—H9	106.5	C24—C23—H23	119.9
O3—C10—O4	123.72 (15)	C22—C23—H23	119.9
O3—C10—C9	126.64 (14)	C25—C24—C23	119.84 (18)
O4—C10—C9	109.64 (13)	C25—C24—H24	120.1
O4—C11—H11A	109.5	C23—C24—H24	120.1
O4—C11—H11B	109.5	C24—C25—C26	120.33 (18)
H11A—C11—H11B	109.5	C24—C25—H25	119.8
O4—C11—H11C	109.5	C26—C25—H25	119.8
H11A—C11—H11C	109.5	C25—C26—C21	120.42 (17)
H11B—C11—H11C	109.5	C25—C26—H26	119.8
C13—C12—C17	118.96 (13)	C21—C26—H26	119.8
C13—C12—C1	121.05 (13)		
			
C9—N1—C1—C12	163.45 (12)	C1—N1—C9—C10	65.95 (16)
C20—N1—C1—C12	−64.73 (15)	C7—C8—C9—N1	49.38 (16)
C9—N1—C1—C2	40.77 (16)	C7—C8—C9—C10	−79.63 (15)
C20—N1—C1—C2	172.59 (11)	C11—O4—C10—O3	3.4 (2)
N1—C1—C2—C7	−10.29 (17)	C11—O4—C10—C9	−177.57 (15)
C12—C1—C2—C7	−131.08 (14)	N1—C9—C10—O3	−28.2 (2)
N1—C1—C2—C3	171.92 (11)	C8—C9—C10—O3	97.86 (18)
C12—C1—C2—C3	51.13 (17)	N1—C9—C10—O4	152.77 (13)
C7—C2—C3—C4	−0.7 (2)	C8—C9—C10—O4	−81.16 (15)
C1—C2—C3—C4	177.19 (12)	N1—C1—C12—C13	125.53 (14)
C18—O1—C4—C3	0.9 (2)	C2—C1—C12—C13	−111.68 (14)
C18—O1—C4—C5	−177.87 (13)	N1—C1—C12—C17	−53.04 (16)
C2—C3—C4—O1	−178.91 (14)	C2—C1—C12—C17	69.75 (16)
C2—C3—C4—C5	−0.1 (2)	C17—C12—C13—C14	0.0 (2)
C19—O2—C5—C6	−12.4 (2)	C1—C12—C13—C14	−178.56 (13)
C19—O2—C5—C4	167.07 (13)	C12—C13—C14—C15	−0.1 (2)
O1—C4—C5—O2	0.83 (18)	C13—C14—C15—C16	0.1 (2)
C3—C4—C5—O2	−178.07 (12)	C14—C15—C16—C17	0.1 (2)
O1—C4—C5—C6	−179.67 (13)	C15—C16—C17—C12	−0.2 (2)
C3—C4—C5—C6	1.4 (2)	C13—C12—C17—C16	0.2 (2)
O2—C5—C6—C7	177.51 (12)	C1—C12—C17—C16	178.75 (14)
C4—C5—C6—C7	−1.9 (2)	C9—N1—C20—C21	−64.04 (15)
C3—C2—C7—C6	0.19 (19)	C1—N1—C20—C21	162.04 (12)
C1—C2—C7—C6	−177.59 (12)	N1—C20—C21—C22	115.75 (16)
C3—C2—C7—C8	−179.65 (13)	N1—C20—C21—C26	−61.09 (19)
C1—C2—C7—C8	2.57 (19)	C26—C21—C22—C23	0.5 (3)
C5—C6—C7—C2	1.1 (2)	C20—C21—C22—C23	−176.41 (16)
C5—C6—C7—C8	−179.02 (13)	C21—C22—C23—C24	0.7 (3)
C2—C7—C8—C9	−22.20 (18)	C22—C23—C24—C25	−1.2 (3)
C6—C7—C8—C9	157.96 (12)	C23—C24—C25—C26	0.6 (3)
C20—N1—C9—C8	165.87 (12)	C24—C25—C26—C21	0.6 (3)
C1—N1—C9—C8	−61.53 (16)	C22—C21—C26—C25	−1.1 (3)
C20—N1—C9—C10	−66.65 (15)	C20—C21—C26—C25	175.85 (17)

### Hydrogen-bond geometry (Å, °)

**Table:**

*D*—H···*A*	*D*—H	H···*A*	*D*···*A*	*D*—H···*A*
C19—H19A···Cg^i^	0.98	2.82	3.639 (2)	148

Symmetry codes: (i) *x*+1, *y*+1, *z*−1.
